# Supplementary material for: Effectiveness and Safety of Direct Oral Anticoagulants versus Vitamin K Antagonists for People Aged 75 Years and over with Atrial Fibrillation: A Systematic Review and Meta-Analyses of Observational Studies
Source: J Clin Med. 2019 Apr 24;8(4):554. doi: 10.3390/jcm8040554 (PMC6518135; doi:10.3390/jcm8040554)
Supplement: Supplementary file 1 [file jcm-08-00554-s001.zip › Supp_material/JOCM_tableS1_submitted.pdf]

Study details<br>Total participants aged ≥ 75 (percentage of total population)	Data source	Population/Follow-up details	Time free of OAC prior to study entry (months)	Drugs studied	Exposure Period<br><br>Average follow-up duration (years)	Factors reported as being adjusted for/matched by
**Graham et al (2014)<br>USA**<br><br>**79 304 (59%)**	Medicare	Anticoagulant naïve new users.<br>*Index date*: First OAC prescription fill<br>*Censored at*: disenrollment from health plan, study outcome, med supply gap ≥ 3 days, switch to other OAC, initiation of dialysis, admission to NH	Excluded anyone with previous OAC use	dabigatran (AD), warfarin	Oct 2010 – Dec 2012<br>NR	Sociodemographic characteristics, baseline medications, baseline comorbidities, prescriber characteristics, h/o falls, fractures, syncope, walker use, smoking status
**Norby et al (2017)<br>USA**<br><br>**56 807 (42%)**	Truven Health MarketScan Commercial Claims and Encounters database, Medicare Supplemental and coordination of benefits database	New users<br>*Index date:* First OAC prescription fill (new users), first rivaroxaban fill (switchers)<br>*Censored at:* outcome event, health plan disenrollment, end of follow up	3	dabigatran (AD), rivaroxaban (AD), warfarin	Jan 2010 – Dec 2014<br>1 (mean, new users)	Age, sex, time since database enrolment, drug initiation date, high dimensional propensity scores included empirical and pre-defined variables (age, age >= 75, calendar year, sex, CHA_2DS_2-VASC score, any prevalent outcome before the start of the index date, baseline comorbidities, baseline medications).
**Avgil-Tsadok et al (2016)<br>Canada**<br><br>**42 478 (67%)**	Provinical hospital discharge database linked to Provincial physician and prescription database	New/prevalent users. Patients could be new to anticoagulation, existing warfarin users or switch from warfarin to dabigatran.<br>Index date: First OAC prescription fill<br>Censored at: outcome event, death, end of follow up	None	dabigatran (AD) warfarin	Jan 1999 – Mar 2013<br>1.3 (median)	Matched on date of 1st dabigatran prescription, year of AF index diagnosis. Propensity scores calculated using: co-morbidities, type of AF diagnosis (principal or secondary), use of other medications, and time to first dabigatran or matched warfarin prescription since AF index hospitalisation)

Study details Total participants aged ≥ 75 (percentage of total population)	Data source	Population/Follow-up details	Time free of OAC prior to study entry (months)	Drugs studied	Exposure Period<br><br>Average follow-up duration (years)	Factors reported as being adjusted for/matched by
**Friberg et al (2017) Sweden**<br><br>**33 597 (49%)**	National Swedish Patient Register, dispensed drug register, socioeconomic longitudinal integration database for health insurance and labour market (LISA)	Anticoagulant naïve new users<br>Index date: First OAC purchase<br>Censored at: First purchase of another OAC, outcome event, end of study period	Excluded anyone with previous OAC use	dabigatran (AD), rivaroxaban (AD), apixaban (AD), warfarin	Dec 2011 – Dec 2014<br>DOAC: 0.71<br>VKA: 1.74<br>(median in mortality group)	Propensity scores calculated using: sociodemographic characteristics, newly diagnosed AF, any previous ICH, any other major bleeding event, baseline comorbidities and medication use.
**Li X et al (2017) USA**<br><br>**31,204 (41%)**	Truven MarketScan, Medicare Supplemental and Coordination of Benefits Database ("MarketScan")	New users<br>Index date: Date of first OAC claim<br>Censored at: 1 year post index date, >30 day prescription gap, outcome event, prescription for alternative OAC, death, end of continuous health plan enrolment, end of follow up	12	apixaban (AD), warfarin	Jan 2012 – Sept 2015<br>DOAC: 0.43<br>VKA: 0.45<br>(mean)	Propensity scores calculated using: age, gender, geographic region, Charlson co-morbidity score, baseline bleeding and stroke/SE history, comorbidities and baseline medications.

Study details<br>Total participants aged ≥ 75 (percentage of total population)	Data source	Population/Follow-up details	Time free of OAC prior to study entry (months)	Drugs studied	Exposure Period<br><br>Average follow-up duration (years)	Factors reported as being adjusted for/matched by
**Nielsen et al (2017) Denmark**<br><br>**29,206 (52%)**	Danish national prescription registry; Danish civil registration system; Danish national patient register	New users<br>Index date: First OAC prescription claim<br>Censored at: outcome event, death, emigration, end of follow up	12	dabigatran (LD) apixaban (LD), rivaroxaban (LD), warfarin	Aug 2011 – Apr 2016<br>2.3	Propensity score models included: age (continuous), binary indicators for sex, hospital diagnosis of AF, baseline co-morbidities, baseline medication, CHA2DS2-VASC and HAS-BLED scores were included as continuous variables.
**Lauffenburger et al (2015) USA**<br><br>**26,867 (41%)**	Truven Health MarketScan, Medicare Supplement Databases	New users<br>Index date: First OAC prescription fill<br>Censored at: loss of continuous health plan eligibility, outcome event, end of follow up	12	dabigatran (AD), warfarin	Oct 2010 – Dec 2012<br>1.01 (mean)	Propensity scores incorporated age, sex, region, insurance plan, generosity of benefit plan, co-morbidities, CHA2DS2-VASC, ATRIA, medication use.
**Seeger et al (2015) USA**<br><br>**24,885 (34%)**	MarketScan, Truven and Clinformatics	New users<br>Index date: First OAC dispensing date<br>Censored at: Gap of >14 days from end of last OAC supply, switch to another OAC, NH admission, death, disenrollment from health plan, end of study period	12	dabigatran (AD), warfarin	Oct 2010 – Dec 2012<br>0.38	Pre-defined investigator specified co-variates including: age, sex, census region, calendar time (by day in 6 month block), co-morbidities, concomitant medications, number of medications, number of hospitalisations, number of days in hospital, number of office visits, number of cardiologist visits, number of neurologist visits, hospitalisation in 30 days prior to treatment initiation, number of lab tests ordered, number of INR tests ordered, number of lipid tests ordered, number of creatinine tests ordered, treating prescriber.

Study details Total participants aged ≥ 75 (percentage of total population)	Data source	Population/Follow-up details	Time free of OAC prior to study entry (months)	Drugs studied	Exposure Period<br><br>Average follow-up duration (years)	Factors reported as being adjusted for/matched by
**Maura et al (2015) France**<br><br>**17,966 (55%)**	The French National Health Insurance information system (SNIIRAM)	New users<br>Index date: First reimbursement of OAC<br>Censored at: index date plus 90 days, >2 consecutive months with no reimbursement, end of year of inclusion, end of follow up	24	dabigatran (AD), rivaroxaban (AD), any VKA (majority fluindione)	Jul 2011 – Nov 2011<br>0.25	Age at index (as a categorical variable), deprivation index of patients municipality of residence, speciality of first OAC prescriber, co-morbidities or co-medications deemed to affect risk of bleeding or arterial thromboembolic events
**Adeboyeje et al (2017) USA**<br><br>**17,521 (40%)**	HealthCore Integrated Research Environment (HIRE)	New users<br>Index date: First OAC prescription fill<br>Censored at: >45 days after a 30 day prescription fill, switch to another OAC, health plan disenrollment, death, end of follow up	6	dabigatran (AD), apixaban (AD), rivaroxaban (AD), warfarin	Nov 2010 – Feb 2015<br>NR	Confounding factors controlled for included: sociodemographics, Deyo-Charlson Comorbidity Index, CHA2DS2-VASC score), modified HAS-BLED, (labile INR not included), comorbidities and medication at baseline hospitalisation before treatment initiation
**Halvorsen et al (2017) Norway**<br><br>**16,034 (49%)**	Norwegian patient registry and Norwegian prescription database.	New users<br>Index date: First OAC prescription fill<br>Censored at: >30 days from calculated end of OAC supply, switched OAC, death, end of follow up	6	dabigatran (AD), apixaban (AD), rivaroxaban (AD), warfarin	Jan 2013 – Jun 2015<br>0.43 – 0.58 (median)	Age, gender, previous bleeding, previous OAC use, co-morbidities and concomitant medications at baseline.

Study details Total participants aged ≥ 75 (percentage of total population)	Data source	Population/Follow-up details	Time free of OAC prior to study entry (months)	Drugs studied	Exposure Period<br><br>Average follow-up duration (years)	Factors reported as being adjusted for/matched by
**Abraham et al (2015) USA**<br><br>**13,845 (38%)**	Optum Labs Data Warehouse	New users<br>Index date: First filled OAC prescription<br>Censored at: gap of 30 days between prescriptions, outcome event, disenrollment from health plan, termination of OAC (>45 days since last prescription fill)	12	dabigatran (AD), rivaroxaban (AD), warfarin	Nov 2010 – Dec 2013<br>NR	Propensity scores included: Risk factors for gastrointestinal bleeding; race; age categories (18-64, 65-75, >=76); drug classes; controlled for follow-up times by including a categorical variable representing the quarter in which the OAC was started.
**Cha et al (2017) South Korea**<br><br>**11,873 (27%)**	National Health Insurance Service (NHIS) database	New users (excluded people with OAC prescription in 2013)<br>Index date: First OAC prescription<br>Censored at: outcome event, emigration, death, end of follow up	≥ 12	dabigatran (AD), rivaroxaban (AD), apixaban (AD), warfarin	Jan 2014 – Dec 2015<br>DOAC: 0.5<br>VKA: 1.51	Propensity score not well described, only appears to contain CHA2DS2-VASC score. No other factors reported as being adjusted for.
**Chan YH et al (2016) Taiwan**<br><br>**11,713 (60%)**	National Health Insurance database (NHIRD)	New and prevalent users? (Description unclear)<br>Index date: First OAC prescription after Jun 2012<br>Censored at: outcome event, end of study period	None	dabigatran (AD), warfarin	Jun 2012 – Dec 2013<br>DOAC: 0.67<br>VKA: 0.73 (median)	Covariates included in propensity score: risk factors for bleeding and cardiovascular events, use of medication at baseline, history of bleeding and hospitalisation.

Study details Total participants aged ≥ 75 (percentage of total population)	Data source	Population/Follow-up details	Time free of OAC prior to study entry (months)	Drugs studied	Exposure Period<br><br>Average follow-up duration (years)	Factors reported as being adjusted for/matched by
**Forslund et al (2017) Sweden**<br><br>**10,753 (48%)**	Administrative health data from the Stockholm region	New users<br>Index date: First claim for OAC<br>Censored at: primary endpoints, migration out of region, switch OAC or change to aspirin, end of follow up	18	dabigatran (AD), apixaban (AD), rivaroxaban (AD), Warfarin	Jan 2012 – Dec 2015<br>DOAC: 1.07<br>VKA: 1.61 (mean)	The composite effectiveness endpoint was adjusted for: individual CHA2DS2-VASC criteria with age as a continuous variable. Severe bleeds adjusted for: sex and adapted HAS-BLED (anaemia, severe bleed, TIA/stroke, liver disease, renal disease, alcoholism, and prior antiplatelet therapy) with age as a continuous variable; Further analyses were done fully adjusted for numerous co-morbidities
**Lau et al (2017) Hong Kong**<br><br>**3978 (48%)**	Clinical Data Analysis and Reporting System (CDARS) of the Hong Kong Hospital authority	New users<br>Index date: First OAC prescription<br>Censored at: end of study period, switch to alternative OAC, discontinuation of OAC (>5 day gap between consecutive prescription refills)	6	dabigatran (AD), warfarin	Jan 2010 – Sept 2015<br>1.16 (mean)	Propensity scores used the following: age, sex, index year, number of hospitalisations within 1 year prior to index date, comorbidities, history of fall, Charlson comorbidities index, and recent use (<=90 days prior to index date) of specified medications.
**Chan PH et al (2016) Hong Kong**<br><br>**571 (100%)**	Hospital AF registry (Queen Mary Hospital)	New and prevalent users? (Unclear from description – states OAC naïve but not stated how this was verified)<br>Index date: First occurrence of AF<br>Censored at: Not reported	NR	dabigatran (LD), warfarin	Jan 2010 – Dec 2013<br>2.6 (mean – ischemic stroke)	None reported.

Study details<br>Total participants aged ≥ 75 (percentage of total population)	Data source	Population/Follow-up details	Time free of OAC prior to study entry (months)	Drugs studied	Exposure Period<br><br>Average follow-up duration (years)	Factors reported as being adjusted for/matched by
**Kwon et al (2016)<br>South Korea**<br><br>**293 (100%)**	Hospital database (Asan Biomedical Research Environment)	New and prevalent users? Not described<br>Index date: Not described<br>Censored at: Not described	NR	dabigatran (AD), rivaroxaban (AD), warfarin	Jan 2011 – Sept 2014<br>DOAC: 0.91<br>VKA: 1.07<br>(Mean)	Sex, anticoagulant type, concomitant antiplatelet use, HAS-BLED score.
**Bengtson et al (2017)<br>USA**<br><br>**Total no. participants: 145 666<br>(≥ 75 years NR)**	Truven Health MarketScan commercial claims and the Medicare Supplemental and Coordination of Benefits database.	Incident DOAC users – classified initially as DOAC users then as OAC naïve or switchers based on previous warfarin use<br>Index date: First OAC prescription<br>Censored at: health plan disenrollment, end of follow up	NR	dabigatran (AD), rivaroxaban (AD), warfarin	Jan 2009 – Dec 2012<br>1.25<br>(median)	Matched on: age (+/- 3 years), sex, time since database enrolment. High dimensional propensity scores (HDPS) calculated using: age, sex, inpatient diagnostic codes, inpatient procedure codes, outpatient diagnostic codes, outpatient pharmacy claims. For each outcome the cox proportional hazards models were adjusted for HDPS decile as well as age, sex, and CHADS2 score.

Study details Total participants aged ≥ 75 (percentage of total population)	Data source	Population/Follow-up details	Time free of OAC prior to study entry (months)	Drugs studied	Exposure Period<br><br>Average follow-up duration (years)	Factors reported as being adjusted for/matched by
**Go et al (2017) USA**<br><br>**Total no. participants: 50 578 (≥ 75 years NR)**	Sentinel Distributed Database	New users<br>Index date: First dispensing of OAC<br>Censored at: treatment discontinuation, switch to alternative OAC, NH admission, disenrollment, death	12	dabigatran (AD), warfarin	Nov 2010 – May 2014<br>DOAC: 0.34<br>VKA: 0.28 (mean)	Propensity score included: Age, sex, indicators of health service use, medical history, recent procedures, frailty indicators, medication use.
**Nishtala et al (2016) New Zealand**<br><br>**Total no. Participants: 10 090 (≥ 75 years NR)**	National Minimum Dataset (NMDS)	New users<br>Index date: First OAC prescription<br>Censored at: outcome event, cessation of OAC (>30 days between refills), death, end of follow up	18	dabigatran (AD except 75mg), warfarin	Jul 2011 – Dec 2012<br>NR	Factors incorporated in to the propensity score are not stated. Matched on age, sex, ethnicity, medical conditions, medication use and chronic disease score

Study details Total participants aged ≥ 75 (percentage of total population)	Data source	Population/Follow-up details	Time free of OAC prior to study entry (months)	Drugs studied	Exposure Period<br><br>Average follow-up duration (years)	Factors reported as being adjusted for/matched by
**Hernandez et al (2015) USA**<br><br>**Total no. participants: 9404 (≥ 75 years NR)**	Centers for Medicare and Medicaid Services (CMS)	New users<br>Index date: First OAC prescription<br>Censored at: cessation of OAC (>60 days between refills), switch to alternative OAC, death, end of follow up.	NR	dabigatran (AD), warfarin	Oct 2010 – Dec 2011<br>DOAC: 0.48<br>VKA: 0.62<br>(mean)	Propensity scores included: age, sex, race, Medicaid eligibility, CHADS2 score, co-morbidities and medication, history of hospitalisation, no. of CMS priority comorbidities, concurrent risk score (higher values predict greater medical spending).

Table S1. Description of included studies. Studies are ordered by size from largest to smallest number of people aged ≥ 75 years. NR = Not reported, AD = any dose, LD = low dose, NH = nursing home, OAC = oral anticoagulant, DOAC = direct oral anticoagulant, VKA = vitamin K antagonist, CHA2DS2-VASC = stroke risk calculator for AF patients (congestive heart failure, hypertension, age ≥ 75, diabetes mellitus, history of stroke/TIA/thromboembolism, vascular disease, age 65-74, female sex), HAS-BLED = bleeding risk score (hypertension, abnormal renal/liver function, stroke, bleeding, labile international normalised ratio (INR), elderly (≥ 65), drugs or alcohol), ATRIA = bleeding risk score (anaemia, severe renal disease, age ≥ 75, prior haemorrhage, hypertension history). Note average follow up duration and exposure period is reported for the overall population not just people aged ≥ 75 years.
